# Crofton Risk and Relative Transactional Entropy

**DOI:** 10.3390/e28020244

**Published:** 2026-02-20

**Authors:** Marcin Makowski, Edward W. Piotrowski

**Affiliations:** 1 Faculty of Physics, Department of Mathematical Methods in Physics, University of Białystok, ul. Ciołkowskiego 1L, 15-245 Białystok, Poland; 2 Independent Researcher, 15-667 Białystok, Poland

**Keywords:** risk, annual percentage rate (APR), entropy, trajectory temperature, complex system, Radon transform, financial instrument, market

## Abstract

We develop a geometric approach to financial risk based on Crofton’s idea and the tools of the Radon transform. The trajectory of a financial instrument is defined with respect to a frame of reference (money, benchmark). A central role is played by simple instruments, inspired by the annual percentage rate (APR) concept, whose graphs in a fixed reference frame are line segments. Risk is interpreted transactionally as the density of exchange dilemmas that arise when the instrument’s trajectory intersects the trajectories of simple instruments. This perspective leads to a risk measure given by the trajectory length in the Crofton–Steinhaus sense. We also introduce new notions, such as geometric volatility, transactional entropy, and trajectory temperature, associated with the distribution of the number of intersections, enabling thermodynamic analogies to be incorporated into the description of risk and market complexity.

## 1. Introduction

In [1], a new measure of risk was proposed, whose key assumption is a geometric distinction between the trajectories of the rates of return of financial instruments. In this model, a special role is played by the so-called simple instruments. Their representation, in a fixed frame of reference based on two distinguished base instruments of a currency-like nature, consists of line segments. It is precisely with respect to these instruments that the riskiness of more complex assets is estimated. The key tool here is the Radon transform, which makes it possible to analyze how frequently the instrument’s trajectory is intersected by lines. The more such intersections occur, the more potential points of exchange (buying or selling) there are, and hence the higher the risk. In this way, risk is treated as a function of the number of transactional dilemmas faced by an investor.

With this approach, risk is not reduced solely to volatility; to some extent, it also reflects market liquidity, understood as the ease of exchanging financial instruments. It turns out that this understanding of risk reveals its relationship with the length of the trajectory of a financial instrument and with its entropy.

For years, financial analysts have been examining the price trajectories of financial instruments, searching for patterns that would allow them to predict the future directions in which the market will move. The analysis of geometric properties of price volatility, such as shape, direction, and above all, length, understood as the total path traversed by the price (rate of return) over time, can provide an important indication when determining key market parameters, such as the risk associated with a given financial instrument. The price trajectories of financial instruments are empirical in nature: they are formed on the basis of data provided by the market and recorded at fixed time intervals. They are not the result of any theoretical model, but a direct reflection of market events.

Length is not a continuous functional. In any arbitrarily small neighborhood of a curve, one can modify its shape so as to produce an arbitrarily large increase in its length. We encounter this paradox when analyzing real trajectories of financial-instrument prices, which may be highly jagged and contain small, frequent fluctuations. This is a problem analogous to measuring the length of a coastline, which depends crucially on the resolution of the map used for the measurement and can increase significantly as micro-details of the land are taken into account. To avoid these difficulties, various generalizations of the definition of length have been proposed [2].

One of the more interesting ideas for measuring length is to use probabilistic methods. This idea was proposed by Laplace [3] and developed by Crofton [4], who defined measures on sets of lines in the plane. In turn, a practical method for measuring the length of empirical curves—which became the inspiration for the considerations presented in this article—was proposed by Hugo Steinhaus [5]. This idea is based on Crofton’s approach, known from the classical Buffon’s needle problem concerning the distribution of the number of intersections between a randomly thrown needle and a family of parallel lines. Steinhaus proposed measuring the length of a curve by counting its intersection points with the lines of a randomly overlaid grid, the so-called longimeter [6].

Let us therefore consider a planar curve γ of finite length |γ|. Lines in the plane $R^{2}$ are described by an equation of the form:

ax+by+1=0.

The pair $\left(a,b\right)\in R^{2}$ uniquely determines a line that does not pass through the origin of the coordinate system. Lines passing through the origin can be omitted, since the measure of the set of such lines is equal to zero. On the space of these lines, we define the Lebesgue measure:

(1) dμ:=ρ(a,b)dadb,

where ρ(a,b) is a function that depends on the parametrization of lines and can be chosen so that the measure μ is invariant under geometric transformations such as rotations or translations of the coordinate system.

Let I(γ) denote the set of lines intersecting the curve γ. According to the classical Crofton theorem, there exists a constant κ>0, depending only on the chosen parametrization of lines, such that

(2) |γ|=κ·μ(I(γ)).

Thus, the length of the curve can be identified, up to a constant factor, with the measure of the set of lines that intersect it. On the set I(γ) one can define

$$dp:=\frac{d\mu }{\mu (I(\gamma ))},$$

Since

$$\int_{I(\gamma )}dp=\int_{I(\gamma )}\frac{d\mu }{\mu (I(\gamma ))}=\frac{\mu (I(\gamma ))}{\mu (I(\gamma ))}=1\;,$$

the measure dp defines a probability distribution for selecting a random line intersecting γ.

Formula (2) can be expressed in a less abstract, probabilistic form. Specifically, we normalize the Crofton measure in (2) restricted to the set of lines intersecting γ and interpret it as a probability law for a randomly chosen line conditioned to intersect γ. Let $P_{\theta }\left(k\right)$ denote the probability that such a randomly chosen line intersects γ in exactly *k* points. Then,

$$\sum_{k=1}^{\infty }P_{\theta }\left(k\right)=1,$$

and the expected number of intersections is proportional to the length of the curve

(3) $$\sum_{k=1}^{\infty }k\cdot P_{\theta }\left(k\right)\propto \left|\gamma \right|\;,$$

since (2) states that the Crofton-weighted average of the intersection count #(γ∩𝓁) over lines 𝓁 equals a constant multiple of |γ|. Here θ denotes a parameter (or a vector of parameters) of the distribution of the number of intersections.

### 1.1. Objectives and Contributions

In this paper, we investigate whether the length of a financial instrument trajectory observed in a fixed financial frame of reference can be treated as a carrier of risk interpreted transactionally as the density of exchange dilemmas of the considered instrument into so-called simple instruments. The more such exchange dilemmas occur during the observation period, the higher the risk of losing the instrument as a good, because more often there appears a real possibility of disposing of it in favor of an alternative of lower risk.

We adopt a frame of reference defined by money and a benchmark instrument and define reference time, thereby establishing a financial instrument trajectory. We represent the above intuition geometrically by associating the number of transactional dilemmas with the frequency of intersections between the instrument trajectory and the family of simple instruments, that is, instruments whose trajectories in the chosen frame of reference are line segments. Next, we define risk as the intensity of the trajectory length in the Crofton–Steinhaus sense, using the Radon transform. Since empirical trajectories are often jagged, irregular, and potentially fractal, the classical notion of length increases with growing observation resolution and loses its operational meaning; therefore, we introduce the length of order *m*, which controls this paradox and yields a more stable measure.

The original contribution of the paper is to link risk with the length of a financial instrument trajectory in such a way that the intersection frequency with the family of simple instruments gives this length a clear transactional interpretation. The same construction then allows us to introduce additional quantities describing complexity, in particular relative transactional entropy and trajectory temperature, which organize the relationship between the intensity of transactional dilemmas and the uncertainty of their structure.

It is worth noting that, in our approach, astronomical time appears only implicitly, so the same price or exchange-rate relationships between instruments yield identical risk measurements. In traditional approaches that depend explicitly on time, this property does not occur. In an era when instantaneous market reactions are possible, for example, due to AI, the absence of an explicit dependence of risk on astronomical time may become a fundamental requirement for this indicator. For the same reason, stemming from the lack of time in an explicit form, there is no obstacle to applying our risk measure even to market games conducted on markets of counterfactual scenarios or alternative histories.

The target audience of this article includes econophysicists and complex systems researchers, as well as quantitative finance, applied mathematics, and statistics specialists, interested especially in geometric measures of risk.

### 1.2. Background on Non-Stochastic Risk Measures

The non-stochastic description of risk proposed in this paper, in which risk is expressed by the length of the trajectory of the rate of return of a financial instrument, does not require any probabilistic assumptions. The literature offers several strands in which risk is not reduced to a probabilistic description, but is understood as a property of decisions, perception, or of the observed path itself. In behavioral economics, a key reference point is the prospect theory of Kahneman and Tversky [7], where risk assessment follows from reference points and from asymmetry in the perception of gains and losses, that is, from the valuation mechanism rather than from a probabilistic model. This approach became a lasting component of economics because it allows one to describe behavior under uncertainty without the need to assume a specific random model.

An important contribution to non-stochastic views of risk is provided by work on model uncertainty and decision making under incomplete knowledge. Such studies emphasize the distinction between measurable risk and unmeasurable uncertainty, introduced classically by Knight [8]. In this spirit, robust approaches are developed, where one considers a family of admissible scenarios and analyzes decisions in a cautious variant aimed at minimizing the maximal possible loss [9,10]. This line of reasoning shifts the focus from estimating probability distributions to constructing quantities that remain stable across a whole class of possible scenarios within a specified uncertainty set.

Closest to the idea of a non-stochastic, trajectory-based view of risk are pathwise and probability-free approaches in mathematical finance, where price is treated as an observed path, and conclusions are drawn without imposing a probability law. In this perspective, risk can be linked not to model parameters, but to features of the path itself, such as its geometry, roughness, or quantities defined directly on the trajectory. A representative example is Itô calculus without probability. Föllmer [11] showed that to apply the Itô formula to a single, concrete path, for instance a historical stock price, it suffices to impose purely analytic conditions on that path rather than assume its randomness.

From the critique of probabilistic assumptions in market modeling, the game-theoretic approach of Shafer and Vovk [12] also emerged. In their framework, “probability” is not defined by an a priori measure, but by the construction of trading strategies within a game. A similar direction is taken in Vovk’s work on rough paths in idealized markets [13], which analyzes path roughness and the variation index in a way that is explicitly free of stochastic assumptions.

The literature also stresses that observed price paths are irregular and often exhibit scaling properties. This motivates descriptions that appeal to the geometry of the trajectory and its roughness. Mandelbrot [14,15] already emphasized that price changes can have a nonstandard structure and that their character may manifest in a similar way across different observation scales. Later developments, including Peters’ fractal market hypothesis [16], extend this viewpoint by pointing to the multiscale organization of markets and its consequences for price dynamics. As a result, these observations support the search for risk measures grounded in geometric features of the path, rather than exclusively in a probabilistic description of its changes.

## 2. Paper Organization

The paper presents a non-stochastic perspective in which risk is treated as a geometric property of an instrument trajectory in a fixed frame of reference. It develops this viewpoint into a coherent construction of a risk measure based on trajectory length. It also introduces additional quantities describing market complexity that follow from the same intersection-based framework.

In Section 3, we establish the starting point, namely the frame of reference determined by the pair money and benchmark instrument, together with the corresponding financial time. From these notions, we derive the definition of a financial instrument trajectory and introduce the concept of simple instruments. These simple instruments serve as the reference objects throughout the subsequent analysis.

Next, in Section 4, we justify that simple instruments are not an ad hoc construction. They arise naturally from reducing the information about an instrument to a single parameter, in the spirit of the APR rate. This explains why their trajectories are line segments in the considered frame of reference.

In Section 5, we move from defining simple instruments to ensuring the comparability of results. We do this by constructing an invariant measure on the space of lines that is consistent with the symmetries of the model. This guarantees that the quantities introduced later depend on the geometry of the trajectory rather than on descriptive conventions. This choice is crucial because it allows us, in Section 6, to relate the number of intersections of a trajectory with simple instruments to the trajectory length in the Crofton–Steinhaus sense. We further introduce the length of order *m* to preserve the stability of the construction for empirical trajectories, which are typically irregular and jagged.

On this basis, we define (Section 7) a risk measure as the intensity of length in financial time and interpret it transactionally as the density of potential exchange points of a risky instrument for a simple instrument. Then, in Section 8, we show, within exemplary models for the distribution of the number of intersections, how the expected length of order *m* relates to volatility. In the inverse step, in Section 9, we define the geometric volatility implied by the observed length.

In Section 10, we introduce relative transactional entropy as a measure of uncertainty in the intersection structure with a randomly chosen line. In Section 11, we complete the thermodynamic analogy by defining trajectory temperature as the sensitivity of entropy to changes in the mean number of intersections. We analyze its properties in the low-temperature regime under the assumption of maximum-entropy distributions. The paper concludes with final remarks and possible directions for further research presented in Section 12.

## 3. Trajectories of Rates of Return of Financial Instruments

In order to build the theoretical foundations for further analysis, it is necessary to present a model of a financial instrument trajectory and a special type of instrument that we refer to as simple instruments. We present this foundation below, following [1], where the interested reader can find a detailed discussion of this model.

### 3.1. Financial Frames of Reference and the Graph of a Financial Instrument

Let us refer to the set of financial instruments and denote by *I* all liquid units of goods or liabilities that are priced continuously. This set, therefore, also includes portfolios, which are combinations of specified quantities of financial instruments quoted directly on the market, as well as any liquid derivative instruments.

The valuation of a financial instrument must always refer to a certain quantity of a distinguished good, also an instrument, usually a monetary unit, with respect to which the instrument is valued. We will call this good money. Moreover, in the set *I* let us distinguish an arbitrary financial instrument different from money and call it the benchmark instrument.

A frame of reference is an ordered pair of distinguished instruments, that is, the pair (money, benchmark instrument). The choice of this pair is not an absolute property of the market, but reflects the descriptive convention adopted by the observer and the instrument selected as the benchmark. Consequently, different observers may describe the same financial instruments in different frames of reference, which is a feature rather than a limitation of the proposed model.

Let τ denote astronomical time, measured in arbitrary units (year, week, second). By r(τ) we will denote the function called the instantaneous growth rate of the benchmark instrument relative to money, expressed in a unit that is the inverse of the unit of time τ, given by

(4) $$r\left(\tau \right):=\frac{1}{p_{s}\left(\tau \right)}\frac{dp_{s}\left(\tau \right)}{d\tau }=\frac{d}{d\tau }\ln p_{s}\left(\tau \right),$$

where $p_{s}\left(\tau \right)$ is the price at time τ of the benchmark instrument *s* expressed in monetary units, and ln is the natural logarithm function.

Note that the differentiability assumption for $p_{s}\left(\tau \right)$ used in (4) is an idealization and serves only to write the instantaneous growth rate in the classical sense. It is not necessary. Reference time can be defined directly via the logarithmic increment of the benchmark price *s*,

$$t\left(\tau \right)=\ln p_{s}\left(\tau \right)-\ln p_{s}\left(\tau_{0}\right),$$

which in discrete data corresponds to increments $\Delta t_{i}=\ln p_{s}\left(\tau_{i}\right)-\ln p_{s}\left(\tau_{i-1}\right)$, without referring to a derivative.

It is worth noting that in the classical term structure theory, the same functional form appears for the short rate when a process analogous to $p_{s}$ plays the role of the money-market account, that is, the numeraire, and the corresponding discount factor satisfies the relation $D\left(\tau \right)\propto 1/p_{s}\left(\tau \right)$. In this case, r(τ) can be read as an instantaneous logarithmic rate associated with discounting. We emphasize that in the present work, this resemblance is purely formal (structural) and should not be understood as a full financial analogy, since $p_{s}$ denotes the price of the chosen benchmark instrument *s*, not necessarily an observable money-market or interest-rate instrument.

Let us define the reference time t=t(τ) as the definite integral of the instantaneous growth rate of the benchmark instrument r(τ), taken from some initial moment $\tau_{0}$ (one may assume $\tau_{0}=0$):

(5) $$t\left(\tau \right):=\int_{\tau_{0}}^{\tau }r\left(\tau^{\prime }\right)\;d\tau^{\prime }.$$

This is time-scaled by market activity. Similarly, the valuation of a derivative instrument (e.g., an option) depends on the time to maturity, which is a form of financial time.

The moment $\tau_{0}$ is an arbitrarily chosen but fixed moment of astronomical time, which determines the zero point of reference time, because

$$t\left(\tau_{0}\right)=\int_{\tau_{0}}^{\tau_{0}}r\left(\tau^{\prime }\right)\;d\tau^{\prime }=0.$$

Note that reference time is defined relatively, it depends on the choice of money and the benchmark instrument. If we swap the goods defining money and the benchmark instrument in the frame of reference, then the instantaneous growth rate of the new benchmark instrument relative to the new money will be equal to −r(τ), so the reference time defined by Formula (5) will change its sign. Therefore, we will call such a swap of the roles of the instruments defining the financial frame of reference an inversion of reference time.

For an arbitrary financial instrument with price p(τ), by the instrument trajectory we will mean the curve $\gamma :\left[\tau_{0},\tau_{1}\right]\to R^{2}$ parametrized by astronomical time τ, given by

(6) $$\gamma \left(\tau \right):=\left(t(\tau ),\;y(\tau )\right),\;y\left(\tau \right):=\ln p\left(\tau \right)-\ln p\left(\tau_{0}\right).$$

For simplicity, we will identify a financial instrument with its trajectory in a fixed frame of reference and still refer to it simply as an “instrument”. In this sense, we will treat the set *I* as the set of trajectories of these instruments.

### 3.2. Simple Instruments

There is nothing to prevent us from considering, in addition to trajectories of financial instruments, trajectories of arbitrary goods for which we know the time evolution of their utility functions. Financial instruments, however, have the advantage that they are priced by the market, so the shapes of their graphs are objective, and their prices result from the operation of market mechanisms that are independent of subjective emotions and views.

Let us call the set of simple instruments $I_{S}$ the family of those financial instruments $\gamma_{S}\in I$ whose trajectory in a fixed frame of reference is a line segment described by the equation

(7) a·t+b·y+1=0.

In the above notation, t=t(τ) is the reference time, and y=y(τ) is the second coordinate of the trajectory at time τ.

We also take into account lines passing through the origin of the coordinate system; however, discussing them separately each time reduces the clarity of the considerations and does not affect the final conclusions. It is sufficient to note that the inclusion of this pencil of lines in the properties of interest follows, for example, from the assumption of Euclidean symmetry imposed on the space of graphs of financial instruments observed from the chosen frame of reference [1]. Thus, abstracting from the time parameters characterizing the period of holding a simple instrument by a market participant, we can characterize such an instrument by a pair of numbers representing, in a given frame of reference, its coordinates $\gamma_{S}:=\left(a,b\right)$. Whether a given financial instrument is simple depends on the choice of the frame of reference.

In the adopted formalism, we allow both long and short positions. From the model’s point of view, a portfolio may contain not only assets but also liabilities, which corresponds to negative coefficients (i.e., multiplication by −1) and provides the natural interpretation of a short position. The geometry of this situation is directly visible within the class of simple instruments. We also consider lines corresponding to returns that decrease in the reference time. In particular, the line y+x=0 represents the logarithmic returns of a short position in the benchmark instrument (valued in money). Since the entire construction (in particular, the measure on the space of lines) is defined to be invariant under the symmetries corresponding to orientation reversal (the transition long↔short), allowing short positions requires neither a change of definitions nor any additional assumptions.

In the approach proposed in [1], the risk of holding a financial instrument is understood as the intensity of the relative “reachability” of its graph by the family of simple instruments in a given frame of reference. In other words, abstracting from technical details, whenever the logarithmic rate of return of a financial instrument becomes equal to the rate of return of one of the simple instruments, a transactional dilemma appears. This is a real temptation to exchange the financial instrument for a simple instrument that is free of this risk. Risk is identified with the intensity of occurrence of such dilemmas with respect to the reference time measure |dt|, that is, per unit of the absolute increment of reference time [1].

In other words, reachability can be viewed as a measure of subjective exchange opportunities. That is, it counts how many times within a given period an investor, from their own perspective (defined by the chosen frame of reference), had the opportunity (a dilemma) to exchange a risky instrument for the investor-chosen risk-free instrument (a simple instrument) at the same relative price.

## 4. Why Do Simple Instruments Arise Naturally in the Reference Pair? From APR to Simple Instruments

The idea of simple instruments is not an artificial construct detached from economic realities. From the point of view of a particular investor, in practice, all information about an instrument can be reduced to a single parameter, analogous to an interest rate that, in the APR (annual percentage rate) approach, serves as a measure of equivalence of value over time.

Let us consider a financial instrument, understood broadly as any economic object whose ownership is associated with cash flows over time. This may be a company, a single traded commodity, a package of services, or a financial contract. We assume that for the considered good, its complete deterministic scenario of cash flows over time is known (for example, from a contract or from the definition of the product), which can be expressed by means of two functions on a certain time interval [0,T]:

$$g:\left[0,T\right]\to R_{\geq 0},\;l:\left[0,T\right]\to R_{\geq 0},$$

where

- g(t)≥0 denotes the intensity of inflows (assets) at time *t*;
- l(t)≥0 denotes the intensity of outflows (liabilities) at time *t*;
- all times t∈[0,T] are measured with respect to the same time system.

In this context, the concept of an interest rate can be introduced as a parameter that allows one to compare the values of flows occurring at different moments in time. Under the assumption of continuous compounding, a constant rate r∈R determines how the value of a unit of money changes over time: an amount *C* at time t=0 corresponds to the amount $C\;e^{rt}$ at time t>0, while conversely, an amount *C* at time *t* has the equivalent value $C\;e^{-rt}$ at time 0.

For a given good with scenario g(·), l(·), we are interested in such a rate *r* for which the value of the stream of assets and liabilities discounted to a single moment is balanced. It is convenient to write this in the form:

(8) $$\int_{0}^{T}g\left(t\right)\;e^{-rt}\;dt\;-\;\int_{0}^{T}l\left(t\right)\;e^{-rt}\;dt\;=\;0.$$

The left-hand side of Equation (8) is a function of the rate *r* and describes the difference between the discounted (at time 0) value of all assets and all liabilities associated with the given good. The rate *r* in (8) plays the role of a balancing parameter: it determines such a conversion of value over time for which the sum of discounted assets and liabilities becomes equal. Such a construction has a purely accounting character and is consistent with the tradition of double-entry bookkeeping developed in Venetian accounting (Luca Pacioli).

If a standard flow structure is assumed (as in typical investment–credit contracts), in particular, a nonzero entry cost and the dominance of outflows at the beginning of the horizon and inflows in its later part, then the existence and uniqueness of a solution r≥0 is obtained. At the same time, in a purely formal sense, this parameter may be allowed to be any real number, i.e., r∈R, and in the subsequent considerations, its sign does not play an essential role. The rate *r* provides a synthetic description of the entire flow scenario and accounts for the full history of the studied good, rather than only its value at a single moment. Such a construction is a direct counterpart of the way the APR rate is defined in credit products [17].

If we reduce the knowledge about the history of changes in the rate of return of a financial instrument to a single parameter, then its trajectory in a fixed frame of reference corresponds to a line segment. To this end, we introduce logarithmic price increments (computed relative to the moment $\tau_{0}$):

(9) $$\begin{array}{c} \ln U_{\tau }\left(v_{0},v_{1}\right)-\ln U_{\tau_{0}}\left(v_{0},v_{1}\right):=x\left(\tau \right),\\ \ln U_{\tau }\left(v_{0},v_{A}\right)-\ln U_{\tau_{0}}\left(v_{0},v_{A}\right):=y_{A}\left(\tau \right). \end{array}$$

where $U_{\tau }\left(v_{0},v_{1}\right)$ denotes the price of the benchmark instrument expressed in money $v_{0}$ at time τ, and $U_{\tau }\left(v_{0},v_{A}\right)$ denotes the price of the considered financial instrument *A* in money $v_{0}$.

We understand the parameter *t* as reference time defined in (5). Note that from the definition of x(τ) and from the definition of reference time we obtain

t(τ)=x(τ),

that is, reference time is equal to the logarithmic rate of return of the benchmark instrument relative to money.

All knowledge about the evolution of prices of instrument *A* from the moment $t_{0}=t\left(\tau_{0}\right)$ to the moment t=t(τ) can be reduced to a minimum by interpreting it so that the change in value between the beginning and the end of the considered interval $\left[t_{0},t\right]$ is, in reference time, equivalent to an investment with a constant rate *r*.

However, since every real investment decision is associated with a deterministic entry cost (commission, tax, and similar items), a natural reference point is the effective acquisition cost of the instrument at time $t_{0}$ (that is, at astronomical time $\tau_{0}$). We model this by a constant multiplier $d_{A}>0$ and define

$$U_{\tau_{0}}^{\mathrm{in}}\left(v_{0},v_{A}\right):=d_{A}\;U_{\tau_{0}}\left(v_{0},v_{A}\right),$$

where $d_{A}$ is a feature of the instrument (or the market), known at time $\tau_{0}$ and independent of τ in the considered interval.

The assumption of simplicity of instrument *A* in this sense is that its price at time τ is consistent with continuous compounding with a constant rate *r*, computed from the entry cost at time $\tau_{0}$, that is

$$\begin{array}{cc} U_{\tau }\left(v_{0},v_{A}\right)\; & =U_{\tau_{0}}^{\mathrm{in}}\left(v_{0},v_{A}\right)\;\exp\left(r\;(t\left(\tau \right)-t\left(\tau_{0}\right))\right)\\ \; & =d_{A}\;U_{\tau_{0}}\left(v_{0},v_{A}\right)\;\exp\left(r\;(t\left(\tau \right)-t\left(\tau_{0}\right))\right). \end{array}$$

Since by definition we have $x\left(\tau \right)=\ln U_{\tau }\left(v_{0},v_{1}\right)-\ln U_{\tau_{0}}\left(v_{0},v_{1}\right)=t\left(\tau \right)$ and $x(\tau_{0})=0$, we obtain equivalently

$$U_{\tau }\left(v_{0},v_{A}\right)=d_{A}\;U_{\tau_{0}}\left(v_{0},v_{A}\right)\;\exp\left(r\;x(\tau )\right).$$

Taking logarithms of both sides of the above equality, we obtain

$$\ln U_{\tau }\left(v_{0},v_{A}\right)=\ln U_{\tau_{0}}\left(v_{0},v_{A}\right)+\ln d_{A}+r\;x\left(\tau \right).$$

Subtracting $\ln U_{\tau_{0}}\left(v_{0},v_{A}\right)$ and using the definition of $y_{A}\left(\tau \right)$ from (9), we obtain the equation of a line:

$$y_{A}\left(\tau \right)=r\;x\left(\tau \right)+b_{A},$$

where $b_{A}:=\ln d_{A}$. In particular, for $d_{A}=1$ we have $b_{A}=0$. We treat $d_{A}$ as a known deterministic entry cost, a feature of the instrument or the market fixed at time $\tau_{0}$, while the only quantity that synthesizes information about the dynamics in reference time is the slope *r*.

The history of every financial instrument is complex, yet after reducing this information to a minimum, we obtain a simple instrument whose description is exhausted by a single parameter *r* (balancing the left-hand side of Equation (8)). This parameter synthesizes the entire history of the instrument and the full cost borne by its holder over the time horizon, and not merely with respect to a single moment, which is often a poorly representative glimpse.

In this approach, the trajectory γ arises as a sequence of simple instruments. At each moment *t*, we treat the considered good as momentarily balanced and assign to it a line with slope *r* determined from Equation (8), on which the point (x(t),y(t)) corresponding to that moment lies in the logarithmic scale. It is precisely the variability of this effective parameter *r*, and thus of successive lines, that makes us observe a curve rather than a segment over the full time horizon.

## 5. Invariant Measure on the Space of Lines

The set of simple instruments will be used to define a new measure of risk and entropy of a financial instrument. For the consistency of the theory, the existence of a measure compatible with the natural symmetries of the considered space is crucial, as it ensures the objectivity and comparability of the conclusions.

For the two-dimensional set of lines intersecting the trajectory γ, consider the class of differential measures of the form (1). Any global change of rates of return, consisting of modifying the inclination angle of the simple instruments intersecting γ, as well as a shift of the origin of the coordinate system along the horizontal axis and an exchange of the roles of assets and liabilities, can be described by the following change of variables:

(10) $$\left(\begin{array}{c} t\\ y \end{array}\right)=\left(\begin{array}{cc} \cos(\varphi ) & -\sigma \sin(\varphi )\\ \sin(\varphi ) & \sigma \cos(\varphi ) \end{array}\right)\left(\begin{array}{c} t^{\prime }\\ y^{\prime } \end{array}\right)+\left(\begin{array}{c} \beta \\ 0 \end{array}\right).$$

where this transformation corresponds to the composition of a rotation by the angle φ, a possible reflection (σ=±1), and a translation by the vector (β,0). This change is described by the family of parameters σ,φ,β such that

σ=±1,φ∈[0,2π),β∈R.

**Remark 1.**  *We assume that the sought measure describing the structure of simple instruments should be independent of arbitrary choices of the frame of reference, which follows from the following premises:*

- *Time shifts, markets have no preferred starting moment, so the model should be independent of the chosen initial point of observation.*
- *Changes of the rate of return by a constant value, which corresponds to a rotation of the graph, that is, a change in the inclination angle of all lines representing the instrument. Such a transformation does not affect the geometric structure of the set of simple instruments; it only changes the growth scale in time.*
- *Reflections of the trajectory of a simple instrument with respect to the horizontal axis, that is, switching from a long position to a short position [1], changing the orientation of the graph does not affect its abstract complexity or the amount of information it contains.*

*Without the requirement of invariance under these transformations, the resulting measure would depend on the adopted conventions for presenting simple instruments, losing its meaning in comparative analyses. Preserving invariance guarantees that the measure describes objective internal properties of the set of simple instruments.*

Transformation (10) induces a transformation of the coordinates of a line intersecting γ:

$$\gamma_{S}=\left(a,b\right)⟶\left(a^{\prime },b^{\prime }\right),$$

where the relation between the new and the old coordinates is as follows:

$$a^{\prime }=\frac{a\cdot \cos(\varphi )+b\cdot \sin(\varphi )}{1+a\cdot \beta },\;b^{\prime }=\sigma \cdot \frac{b\cdot \cos(\varphi )-a\cdot \sin(\varphi )}{1+a\cdot \beta }.$$

The Jacobian of this transformation is

$$\frac{\partial (a^{\prime },b^{\prime })}{\partial (a,b)}=\sigma \cdot {\left(1+a\cdot \beta \right)}^{-3},$$

and

$$1+a\cdot \beta =\sqrt{\frac{a^{2}+b^{2}}{a^{\prime 2}+b^{\prime 2}}},$$

so the invariance of the measure

(11) $$d\mu \left(a^{\prime },b^{\prime }\right)=\rho \left(a^{\prime },b^{\prime }\right)\;da^{\prime }\;db^{\prime }={\left(1+a\cdot \beta \right)}^{-3}\cdot \rho \left(a^{\prime },b^{\prime }\right)\;da\;db=\rho \left(a,b\right)\;da\;db=d\mu \left(a,b\right)$$

requires that

(12) $${\left(a^{2}+b^{2}\right)}^{\frac{3}{2}}\;\rho \left(a,b\right)={\left(a^{\prime 2}+b^{\prime 2}\right)}^{\frac{3}{2}}\;\rho \left(a^{\prime },b^{\prime }\right).$$

This, in turn, implies the following Euclidean invariant measure of lines intersecting the curve γ:

(13) $$d\mu \left(a,b\right)={\left(a^{2}+b^{2}\right)}^{-\frac{3}{2}}\;da\;db.$$

It is convenient to parametrize the set of all simple instruments $I_{S}$ by the pair (p,φ), where

- φ∈[0,2π) is the angle of inclination of the line with respect to the *x* axis;
- $p\in R_{+}$ is the distance of the line from the origin of the coordinate system.

In the new coordinates, the equation of the line (7) is

t·cos(φ)+y·sin(φ)=p.

The relationship between the old and the new coordinates is given by

$$a=-p^{-1}\cdot \cos\left(\varphi \right)\;,\;b=-p^{-1}\cdot \sin\left(\varphi \right)$$

where $p^{-1}=\sqrt{a^{2}+b^{2}}$, and the Jacobian of this transformation is as follows:

$$\frac{\partial (a,b)}{\partial (p,\varphi )}=p^{-3}\;.$$

Using (13), we obtain

$$\mu \left(a,b\right)={\left(a^{2}+b^{2}\right)}^{-\frac{3}{2}}\;da\;db=p^{3}\cdot \frac{\partial (a,b)}{\partial (p,\varphi )}\;dp\;d\varphi \;.$$

Therefore,

(14) dpdφ=μ(p,φ).

## 6. Application of the Ideas of Crofton and Steinhaus to Determining the Length of a Financial Instrument Trajectory

A natural complement and development of the approach to risk modeling proposed in [1] is to view risk through the length of the trajectory of a financial instrument. The set of simple instruments makes it possible to define this length in a chosen frame of reference. This is one of the quantities describing the complexity of the price path. In periods of increased volatility or market unrest, the trajectory is more jagged and, therefore, longer, which is consistent with the previously defined risk understood as the density of transactional dilemmas.

Let us define the Dirac function concentrated on the trajectory of a financial instrument:

$$f_{\gamma }\left(x,y\right):=\int_{\tau_{0}}^{\tau_{1}}\delta \left(x-t\left(\tau \right)\right)\;\delta \left(y-y\left(\tau \right)\right)\;d\tau ,$$

where γ(τ)=(t(τ),y(τ)) is a parametrization of the trajectory of the financial instrument and $\left[\tau_{0},\tau_{1}\right]$ is the time interval over which we observe the instrument. Assume that the curve γ is rectifiable, that is, it has finite length.

### Length of Order m

In this new coordinate system (p,φ), we write the Radon transform of the function $f_{\gamma }\left(x,y\right)$ concentrated on the graph of the financial instrument [1]:

(15) $${\overset{ˇ}{f}}_{\gamma }\left(p,\varphi \right)=\int_{-\infty }^{\infty }f_{\gamma }\left(p\cos\varphi -s\sin\varphi ,\;p\sin\varphi +s\cos\varphi \right)\;ds.$$

The formula ${\overset{ˇ}{f}}_{\gamma }\left(p,\varphi \right)$ determines the number of intersections of the line (p,φ) with the curve γ. According to the classical Crofton theorem, the length of a rectifiable curve $\gamma \subset R^{2}$ can be expressed as an integral over all lines intersecting γ:

(16) $$\left|\gamma \right|=\frac{1}{2}\int_{0}^{2\pi }\int_{0}^{\infty }{\overset{ˇ}{f}}_{\gamma }\left(p,\varphi \right)\;dp\;d\varphi .$$

Realized trajectories of stochastic processes are often characterized by high variability and irregularity, and their classical geometric length can be infinite. For this reason, it is necessary to use an appropriately modified notion of length. In [5], Steinhaus proposed introducing the notion of the length of order *m*, which consists in taking into account at most *m* intersections of a line with a curve, leading to the following modification of Formula (16):

(17) $${\left|\gamma \right|}_{m}:=\frac{1}{2}\int_{0}^{2\pi }\int_{0}^{\infty }\min\left({\overset{ˇ}{f}}_{\gamma }\left(p,\varphi \right),\;m\right)\;dp\;d\varphi \;.$$

This, in practice, leaving aside the abstract notion of a measure, simply means restricting the summation in series (3) to the first *m* terms:

(18) $${\left|\gamma \right|}_{m}=\sum_{k=1}^{m}k\cdot P_{\theta }\left(k\right).$$

The above definition of the length of order *m* makes it possible to control the paradox of length in most practical problems, especially when measuring the length of the trajectory of the evolution process of the rate of return of a financial instrument. As already mentioned, these processes are often characterized by a fractal structure or approximate self-similarity when we observe them on shorter and shorter time intervals, which means that their study requires tools that are robust to changes in the observation scale. In finance, the analysis of such structures can provide important insights into market dynamics and potential mechanisms that generate the observed volatility. It is worth emphasizing, however, that introducing the length of order *m* into financial analysis is not merely an abstract mathematical device, but has a practical justification. It reflects the limited ability of market participants to respond to infinitesimally small price fluctuations. The order *m* plays the role of the maximal level of significance of changes, above which the reactions of market participants become negligible.

The financial literature has long revisited the question of the extent to which price trajectories admit a multifractal description. Mandelbrot’s classical observations and subsequent models suggest that market data often exhibit features such as jagged paths, heavy tails of return distributions, and long-range volatility dependence, which may be consistent with an image of approximate self-similarity over certain ranges of time scales [15,18,19]. At the same time, fractality should not be treated as a market axiom. The observed scale dependence is rarely unambiguous and may be visible only in limited frequency windows, outside of which it can weaken or disappear. Nevertheless, many price trajectories possess properties that, at certain scales, can lead to instability of classical geometric measures. In this context, order-*m* length is used in the paper as a scale regulator that limits the contribution of extremely fine fluctuations. In practice, such fluctuations may be irrelevant for investment decision-making. This approach makes the construction more robust to changes in data frequency and closer to the scale at which investment decisions are actually made.

## 7. A Risk Measure Based on the Trajectory Length

Let $\gamma =\{\left(t\left(\tau \right),y\left(\tau \right)\right):\tau \in \left[\tau_{0},\tau_{1}\right]\}$ be the trajectory of a financial instrument in a fixed frame of reference, observed on the reference time interval $\left[t_{0},t_{1}\right]$. The risk measure of a financial instrument induced by the length of the trajectory of its graph can be defined as the functional

(19) $$R_{m}\left(\gamma ;\left[t_{0},t_{1}\right]\right)\;:=\;\frac{1}{|t_{1}-t_{0}|}\;{\left|\gamma \right|}_{m}.$$

The quantity $R_{m}$ determines the rate of growth of the trajectory length in reference time, and we may call it the intensity of Crofton length. This is a reformulation, in terms of length, of the definition of risk known from [1]. In the proposed model, reference time *t* is defined as the integral of the instantaneous growth rate of the benchmark instrument, and it is dimensionless, which ensures the invariance of $R_{m}$ under changes in the scale of astronomical time units. Moreover, it has the following obvious properties, which are consequences of the invariance of the measure (14) with respect to the group of plane isometries:

1. Invariance under shifts along the logarithmic rate of return axis. For any constant c∈R and the trajectory $\gamma_{c}=\left\{\left(t,y+c\right):\left(t,y\right)\in \gamma \right\}$, we have
  $$|\gamma_{c}{|}_{m}={\left|\gamma \right|}_{m}\;\mathrm{and}\;R_{m}\left(\gamma_{c};\left[t_{0},t_{1}\right]\right)=R_{m}\left(\gamma ;\left[t_{0},t_{1}\right]\right).$$
2. Invariance under inversion of reference time. Let $\gamma^{\mathrm{inv}}=\left\{\left(-t,y\right):\left(t,y\right)\in \gamma \right\}$. Then,
  $$|\gamma^{\mathrm{inv}}{|}_{m}={\left|\gamma \right|}_{m}\;\mathrm{and}\;R_{m}\left(\gamma^{\mathrm{inv}};\left[-t_{1},-t_{0}\right]\right)=R_{m}\left(\gamma ;\left[t_{0},t_{1}\right]\right).$$
3. Monotonicity with respect to *m*. If $m_{1}\leq m_{2}$, then ${\left|\gamma \right|}_{m_{1}}\leq {\left|\gamma \right|}_{m_{2}}$. If |γ|<∞, we have
  $$\lim_{m\to \infty }{\left|\gamma \right|}_{m}=\left|\gamma \right|\;\mathrm{and}\;\lim_{m\to \infty }R_{m}\left(\gamma ;\left[t_{0},t_{1}\right]\right)=\frac{\left|\gamma \right|}{|t_{1}-t_{0}|}.$$

For a fixed, empirically given trajectory γ, the order-*m* length ${\left|\gamma \right|}_{m}$ is a deterministic functional (defined as an integral over the space of lines). Probabilistic terminology appears only as an interpretation of this integral and does not introduce stochastic uncertainty into the underlying geometric reality (i.e., the length of the trajectory), but rather provides a clever tool for measuring it. In the later part of the paper, we impose a probabilistic structure on the space of lines, which makes it possible to define entropy and temperature.

The quantity $R_{m}$, defined in (19), should not be interpreted as a replacement for classical volatility (standard deviation), but as a concept that complements the picture of risk associated with a given financial instrument. $R_{m}$ does not require the existence of the second moment and remains well defined also for trajectories of stochastic processes with heavy tails, for which variance estimation can be unstable. The parameter *m* plays the role of a regulator of the significance of changes; it cuts off the impact of fluctuations below the decision scale of a given investor or institution. It suppresses the influence of frequent disturbances (market noise corrected by appropriate investor reactions), which can artificially inflate measures based solely on volatility.

**Figure 1:**
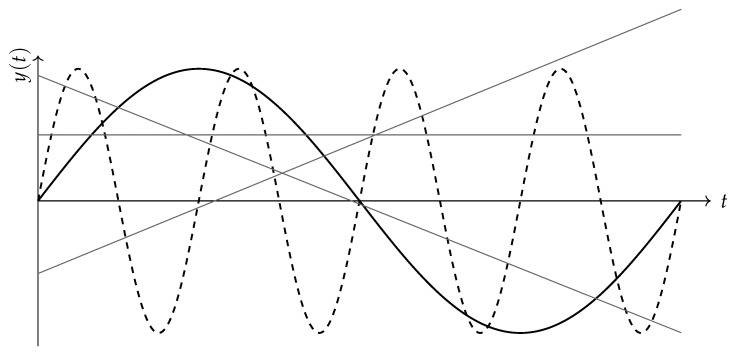
Two trajectories, sin(t) and sin(4t), with the same variance but different lengths. Three sample lines (simple instruments) are shown to illustrate their different numbers of intersections with each curve. This illustrates the geometric intuition behind the Crofton–Steinhaus idea.

Two trajectories with the same variance can have different values of $R_{m}$, see the example in Figure 1. If for the same variance we obtain a larger $R_{m}$, then the trajectory intersects lines more frequently (more local reversals of direction), which means more potential transactions of exchanging the instrument for a simple instrument, and the instrument profile is closer to short-term strategies. A smaller $R_{m}$ for the same variance suggests a smoother path with longer segments of a clear trend. $R_{m}$ distinguishes the geometry of paths with the same intensities of perturbations, providing information complementary to that obtained from variance.

The quantity $R_{m}$ can be interpreted in the context of simple instruments. The more often the trajectory is intersected by lines, the more often potential exchange points appear, and the denser the investor’s decision process becomes. Thus, $R_{m}$ links the notion of risk with liquidity.

It is worth emphasizing that the quantity $R_{m}$ introduced in the paper is not defined as a function of the loss distribution over a prescribed horizon, but as a functional of a trajectory that describes the path length traversed by the price of a financial instrument. Consequently, the classical coherence axioms and elicitability, formulated for risk measures based on a loss random variable [20,21,22], are not a natural point of reference for $R_{m}$, because they concern objects of a different nature. $R_{m}$ is not a risk measure defined on a space of random variables, but a deterministic characteristic of an observed path, and should therefore be treated as a tool complementary to classical measures such as VaR or ES. The quantity $R_{m}$ interprets risk in transactional terms, as a function of the density of potential moments of losing the held financial instrument, understood as the density of transactional dilemmas.

## 8. Trajectory Length and Volatility

Trajectories of rates of return contain information about the direction, scale, and frequency of price fluctuations. The curve length in the Crofton–Steinhaus sense makes it possible to capture those features to which measures based on variance remain insensitive. It is therefore worth examining the dependence of the trajectory length on the standard deviation σ of the process of which it is a realization, which may become an inspiration for defining a new measure of risk.

Assume that the trajectory of a financial instrument γ(t)=(t,Y(t)) is a realization of a stochastic process Y(t) with zero expected value, E[Y(t)]=0, and variance growing linearly in time, $\mathrm{Var}\left[Y\left(t\right)\right]=ct:=\sigma^{2}$. An example of such a process is the Wiener process. Below we analyze two examples of determining the length of order *m* as a function of the process volatility σ, assuming two different distributions for the number of intersections of a line with the trajectory.

### 8.1. Dependence of the Length of Order m on the Process Variance—Poisson Distribution

Assume that for a fixed line (p,φ) the number of intersections ${\overset{ˇ}{f}}_{\gamma }\left(p,\varphi \right)$ is treated as a random variable with a distribution close to the Poisson distribution:

$${\overset{ˇ}{f}}_{\gamma }\left(p,\varphi \right)∼\mathrm{Poisson}\left(\lambda \left(\sigma \right)\right),$$

where the intensity parameter λ(σ) increases with the process variance $\sigma^{2}$. The assumption about this form of the Poisson parameter is consistent with its interpretation in the context of the proposed model. Higher volatility leads to a more jagged trajectory, which results in a larger number of intersections.

Since we are dealing with a random process, we can speak not so much about length as about the expected length of the trajectory γ of a financial instrument, which can be written as follows:

(20) $${E[|\gamma |}_{m}]=\frac{1}{2}\int_{0}^{2\pi }\int_{0}^{\infty }E\left[\min\left({\overset{ˇ}{f}}_{\gamma }\left(p,\varphi \right),m\right)\right]dp\;d\varphi ,$$

Therefore,

$${E[|\gamma |}_{m}]\propto L_{m}\left(\sigma \right):=E\left[\min\left(\mathrm{Poisson}\left(\lambda \left(\sigma \right)\right),m\right)\right]$$

If we assume that the intensity of intersections grows linearly with the standard deviation, λ(σ)=C·σ, where C>0 is a constant depending on the length of the observation time interval, we obtain the following:

$${E[|\gamma |}_{m}]\propto L_{m}\left(\sigma \right)=\sum_{k=0}^{m-1}k\cdot \frac{{\left(C\sigma \right)}^{k}e^{-C\sigma }}{k!}+m\cdot \left(1-\sum_{k=0}^{m-1}\frac{{\left(C\sigma \right)}^{k}e^{-C\sigma }}{k!}\right).$$

The dependence of $L_{m}\left(\sigma \right)$ on the variance $\sigma^{2}$ has intuitive boundary properties:

- For σ→0, low volatility means a small number of intersections, so
  $$L_{m}\left(\sigma \right)∼C\cdot \sigma .$$
- For σ→∞, the number of intersections exceeds *m*, so
  $$L_{m}\left(\sigma \right)\to m.$$

**Figure 2:**
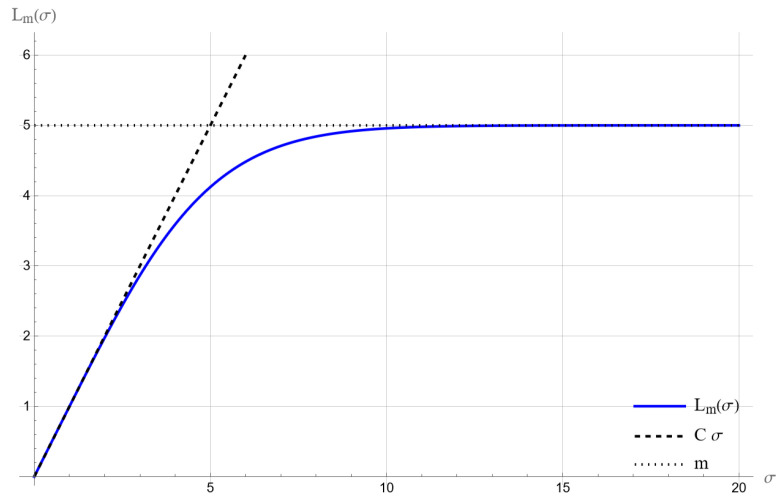
Function $L_{m}$ for the Poisson distribution, m=5.

These dependencies are illustrated in Figure 2.

### 8.2. Dependence of the Length of Order m on the Process Variance—Negative Binomial Distribution

Let us now consider the case in which the number of intersections of the trajectory with the line (p,φ) has a negative binomial distribution:

$${\overset{ˇ}{f}}_{\gamma }\left(p,\varphi \right)∼\mathrm{Negative}-\mathrm{binomial}\left(r,p\left(\sigma \right)\right),$$

where r>0, and p(σ)∈(0,1] is a function depending on the volatility σ. The expected value of this distribution equals

$$r\frac{1-p(\sigma )}{p(\sigma )}.$$

We assume that the expected number of intersections grows linearly with the standard deviation σ, that is

(21) $$r\frac{1-p(\sigma )}{p(\sigma )}=C\cdot \sigma ,$$

where C>0 is a constant depending on the length of the observation time interval. Transforming Equation (21), we obtain

(22) $$p\left(\sigma \right)=\frac{r}{r+C\sigma }$$

The formula for $L_{m}\left(\sigma \right)$ is as follows:

(23) Lm(σ)=∑k=0m−1k·k+r−1k[p(σ)]r[1−p(σ)]k+m·1−∑k=0m−1k+r−1k[p(σ)]r[1−p(σ)]k.

The boundary properties of $L_{m}\left(\sigma \right)$ are identical to the Poisson case.

For the negative binomial distribution, the function $L_{m}\left(\sigma \right)$ reaches the value *m* more slowly than for the Poisson distribution. This follows from the fact that the negative binomial distribution has a variance greater than its mean, which means a larger dispersion of outcomes. In contrast, in the Poisson distribution, the variance equals the mean, so the probability of obtaining a count greater than or equal to *m* increases faster with the growth of the parameter σ, and the function $L_{m}\left(\sigma \right)$ reaches the value *m* faster.

Note that in the proposed model, the expected length of order *m* of the trajectory of a financial instrument $E[|\gamma {|}_{m}]$ is proportional to the function $L_{m}\left(\sigma \right)$. The constant proportionality factor follows from the Crofton integral (20) over all lines and does not affect the conclusions presented below. Therefore, to simplify the exposition, we will consider $L_{m}\left(\sigma \right)$ as a representative of the change in the expected length as a function of volatility σ.

## 9. Geometric Volatility

For a continuous, strictly increasing function $L_{m}\left(\sigma \right)$ and any empirically observed value 𝓁, we define the geometric volatility implied by the length of order *m* as

(24) $$\sigma_{\mathrm{geom}}^{\left(m\right)}\left(𝓁\right)\;:=\;L_{m}^{-1}\left(𝓁\right).$$

This construction is a formal analogue of implied volatility in the Black–Scholes model, where the market option price C is mapped to the parameter $\sigma =f^{-1}\left(C\right)$. In the approach proposed above, the “price” is replaced by the observed length of order *m*. Inverting the function $L_{m}$ provides the parameter $\sigma_{\mathrm{geom}}^{\left(m\right)}$, which we may interpret as a market implied level of risk measured by the geometry of the trajectory rather than by the distribution of rates of return. Finding $\sigma_{\mathrm{geom}}^{\left(m\right)}$ requires numerical methods such as the bisection method, the Newton–Raphson method, or optimization algorithms, for example genetic algorithms.

Let us consider the following example, which is illustrative and helps explain the key aspects of $\sigma_{\mathrm{geom}}^{\left(m\right)}$, abstracting from the complexity of real data.

**Example 1**  (Geometric volatility for the trajectories sint and sin(4t))**.** *Assume that we study two financial instruments whose trajectories are given by*

$$\gamma_{1}\left(t\right)=\left(t,y_{1}\left(t\right)\right),\;y_{1}\left(t\right)=\sin t,\;\gamma_{4}\left(t\right)=\left(t,y_{4}\left(t\right)\right),\;y_{4}\left(t\right)=\sin\left(4t\right),$$

*on the reference time interval t∈[0,2π]. Assuming the negative binomial model with C=1 and r=2, let us determine the length of order m=8 for each of these trajectories and the geometric volatility implied by this model.*

Both trajectories above are equally dispersed around their mean value, which is zero, so they have the same classical volatility:

$$\sigma_{0}:=\sigma_{\left(\gamma_{1}\right)}=\sigma_{\left(\gamma_{4}\right)}=\sqrt{\frac{1}{2}}\approx 0.7071.$$

For the curve $\gamma_{4}$, only the line y=0 intersects this trajectory more than 8 times, namely 9 times. The line y=0 is not distinguished in our construction and is mentioned here only as a convenient illustrative case. For the curve $\gamma_{4}$, it yields nine intersections; however, this concerns in fact a single line, hence a measure-zero subset in the space of all lines. Consequently, it does not contribute to the Crofton integral defining ${\left|\gamma \right|}_{m}$ and may be ignored. For both trajectories considered, the number of intersections with any line is not greater than 8. Therefore, the length of order 8 of these curves equals their classical length

$${\left|\gamma_{k}\right|}_{8}=\left|\gamma_{k}\right|=\int_{0}^{2\pi }\;\sqrt{1+{\left(\frac{d}{dt}\sin\left(kt\right)\right)}^{2}}\;dt$$

Therefore,

(25) $$|\gamma_{1}{|}_{8}=7.64039558,\;{\left|\gamma_{4}\right|}_{8}=17.62855113.$$

From Crofton’s theorem, it follows that there exists a constant κ>0 such that

$${\left|\gamma \right|}_{m}\;\;=\;\kappa \;L_{m}\left(\sigma \right),$$

where $L_{m}\left(\sigma \right)$, under the assumed negative binomial distribution, is given by (23). Let us choose the constant κ so that the geometric volatility of the trajectory $\gamma_{1}$ equals the classical volatility $\sigma_{0}$:

(26) $$\kappa \;L_{8}\left(\sigma_{0}\right)\;=\;{\left|\gamma_{1}\right|}_{8}.$$

Substituting into (23) the value $\sigma_{0}=\frac{1}{\sqrt{2}}$, with r=2, C=1, we obtain

$$L_{8}\left(\sigma_{0}\right)\approx 0.7070461789.$$

Using the length of the curve $\gamma_{1}$ in (25), we have

(27) κ≈10.8060771807.

From the above calibration of the model, we immediately obtain

(28) $$\sigma_{\mathrm{geom}}^{\left(8\right)}\left(\right|\gamma_{1}{|}_{8})=\sigma_{0}=\frac{1}{\sqrt{2}}\approx 0.7071067812.$$

Determining $\sigma^{*}$ from the equation

(29) $$\kappa \;L_{8}\left(\sigma^{*}\right)\;=\;{\left|\gamma_{4}\right|}_{8},$$

where κ and $|\gamma_{4}{|}_{8}$ are the values from (27) and (25), respectively, we obtain

(30) $$\sigma^{*}\;=\;\sigma_{\mathrm{geom}}^{\left(8\right)}\left(\gamma_{4}\right)\;\approx \;1.6402677066$$

The above example illustrates that the Crofton–Steinhaus length records the density of decision points, that is, the frequency of intersections with simple instruments, which is not captured by variance alone. An investor using geometric volatility will distinguish trajectories of financial instruments with the same magnitude of fluctuations but a different frequency of reversals. This has a natural transactional interpretation. More intersections mean more real exchange dilemmas and therefore a higher risk of executing an incorrect transaction. The quantity $\sigma_{\mathrm{geom}}^{\left(m\right)}$ is a practical approach to risk, complementary to classical measures, rooted in the geometry of the trajectory and the intensity of the decision process.

We introduced the Poisson and negative binomial models for the number of intersections as illustrative examples. We do not assume that they provide a universal description of market data. These examples were used to show how the expected length of order *m* can be modeled as a function of a volatility parameter, and to define the geometric volatility $\sigma_{\mathrm{geom}}^{\left(m\right)}$ as a parameter implied by the observed length. The value of $\sigma_{\mathrm{geom}}^{\left(m\right)}$, therefore, depends on the chosen distributional family through the form of the function $L_{m}\left(\sigma \right)$, whereas the length of order *m* itself is a geometric object and does not require probabilistic assumptions. In practice, the distributional family and its parameters can be estimated empirically from market data, based on the distribution of intersection counts obtained by sampling lines from the invariant measure.

## 10. Relative Transactional Entropy of a Financial Instrument

Studying the relationships between trajectory length and risk can be carried out by searching for analogies and methods of analysis taken from the description of physical systems. In particular, introducing into the proposed model the concept of entropy as a measure of the disorder of the trajectory of a financial instrument [23] makes it possible to describe its complexity in a way analogous to the description of thermodynamic systems.

The relationships between thermodynamics, statistical mechanics, and information theory and the analysis of financial markets are intensively studied within a newly distinguished field called econophysics [24,25,26].

Entropy is already a basic tool in the quantitative analysis of financial markets. Approaches such as Jaynes’ maximum entropy principle and constructions based on Tsallis and Rényi entropies have proved useful in describing heavy tails of return distributions, volatility classification, nonlinear critical phenomena and market effects, or option pricing [27,28,29,30,31,32,33,34,35,36,37,38,39,40,41,42,43,44,45]. In the area inspired by quantum formalism, Fisher information is used to study the informational aspect of decision processes in the financial market [46,47,48,49,50,51,52,53,54,55,56].

Within the geometric model considered in this work, a natural carrier of entropy is the distribution of the number of intersections of simple instruments with the trajectory γ of a given financial instrument, that is $P_{\theta }\left(k\right)$, together with the invariant measure of lines intersecting γ. This construction depends on the chosen frame of reference (money, benchmark instrument). Changing the frame changes the family of simple instruments and the distribution $P_{\theta }\left(k\right)$ of the number of intersections (transactional dilemmas) with respect to which we compute entropy. Therefore, we will call it relative transactional entropy.

### 10.1. Invariant Probability Measure

In Section 5 we determined an invariant measure with respect to plane isometries, which allows us to define a probability measure $P_{\gamma }$ on the set of lines intersecting γ.

(31) $$dP_{\gamma }\left(a,b\right):=\frac{{\left(a^{2}+b^{2}\right)}^{-\frac{3}{2}}}{C_{\gamma }}\;da\;db,$$

where the normalizing constant $C_{\gamma }$ is given by

$$C_{\gamma }:=\int_{I(\gamma )}{\left(a^{2}+b^{2}\right)}^{-\frac{3}{2}}\;da\;db.$$

And, analogously,

(32) $$dP_{\gamma }\left(p,\varphi \right):=\frac{1}{C_{\gamma }}\;dp\;d\varphi ,$$

with $C_{\gamma }:=\int_{I(\gamma )}dp\;d\varphi =\mu \;\left(I(\gamma )\right)$ (hence $dP_{\gamma }=d\mu /\mu \left(I\left(\gamma \right)\right)$).

The measure $P_{\gamma }$ is the probability of choosing a random line intersecting the curve γ, consistent with the invariant geometric structure of the space of lines. In particular, for any subset A⊂I(γ)

$$P_{\gamma }\left(A\right)=\mathrm{the}\;\mathrm{probability}\;\mathrm{that}\;a\;\mathrm{random}\;\mathrm{line}\;\mathrm{intersecting}\;\gamma \;\mathrm{belongs}\;\mathrm{to}\;A\;.$$

Introducing the notion of density, we can formulate the above relation as follows:

(33) $$P_{\gamma }\left(A\right)=\int_{A}f\left(a,b\right)\;da\;db.$$

where

$$f\left(a,b\right)=\frac{{\left(a^{2}+b^{2}\right)}^{-\frac{3}{2}}}{C_{\gamma }}.$$

In particular, the function f(a,b) defines a probability distribution on the space of lines intersecting γ.

### 10.2. Entropy

We may introduce the notion of the entropy of the distribution (33), understood as a measure of uncertainty in the choice of a random line intersecting γ:

$$H\left(P_{\gamma }\right)=-\int_{I(\gamma )}f\left(a,b\right)\log f\left(a,b\right)\;da\;db.$$

High entropy indicates that the lines intersecting the curve are distributed in a relatively uniform way in the parameter space (a,b). In other words, there is substantial uncertainty as to which line will be selected by a random process. On the other hand, low entropy means that certain lines in the parameter space are more probable than those that intersect γ.

While differential entropy measures the uncertainty of choosing the line itself that intersects γ, to describe uncertainty about the number of intersection points of this line with the trajectory of a financial instrument, we can refer to Shannon entropy. Let $P_{\theta }\left(k\right)$ denote the probability that a randomly chosen line intersects the graph of a financial instrument $\gamma \subset R^{2}$ in exactly *k* points, $P_{\theta }\left(k\right)=P_{\gamma }\left\{\;𝓁\in I\left(\gamma \right)|N_{\gamma }\left(𝓁\right)=k\;\right\}$. The distribution ${\left\{P_{\theta }\left(k\right)\right\}}_{k\in N_{0}}$ satisfies

$$\sum_{k=0}^{\infty }P_{\theta }\left(k\right)=1.$$

We call the Shannon entropy of the trajectory of a financial instrument γ the quantity

(34) $$S_{\gamma }\left(\theta \right):=-\sum_{k=0}^{\infty }P_{\theta }\left(k\right)\log P_{\theta }\left(k\right),$$

where we adopt the standard continuity convention for the function $S_{\gamma }\left(\theta \right)$:

$$0\log0:=\lim_{x\to 0^{+}}x\log x\;.$$

A high entropy value means greater unpredictability in intersections and thus greater geometric complexity in the trajectory of a financial instrument. For a line segment, which can be treated as a trajectory corresponding to a financial instrument with a constant or linear rate of return, entropy takes particularly low values. The number of intersections is essentially limited to two possibilities: a line does not intersect the segment, or it intersects it in exactly one point. The event in which the segment lies on a line has measure zero. This means that the distribution $\left\{P_{\theta }\left(k\right)\right\}$ is strongly concentrated and predictable, and the probability of an intersection at many points, more than one, equals zero. Geometrically, the segment represents a trajectory of minimal complexity. In contrast, trajectories with a complicated structure, for example, fractal, can be intersected by random lines in many different points, and the number of possible intersection values is obviously much larger than in the case of a segment. As a result, the distribution $\left\{P_{\theta }\left(k\right)\right\}$ becomes more diffuse, and Shannon entropy takes higher values. This means that the structure of such a trajectory is much less predictable. In this sense, entropy is treated as a measure of risk. Note that the above property of entropy is consistent with the risk model given in [1], where simple instruments, that is, those whose trajectories are line segments, are instruments with the lowest level of risk.

## 11. Temperature of a Financial Instrument Trajectory

The thermodynamics-inspired concept of temperature has been introduced into financial analysis and developed independently by many authors. In [57], temperature is an effective scale of money, a constant determined by the average amount of money per agent. In spin models, temperature plays the role of a regulator of decision randomness and market nervousness [58,59]. The so-called social temperature, which changes with the mood of market participants, makes it possible to model feedback between investor behavior and the state of the market [60]. The notion of temperature is also used to assess the quality of an investor’s strategy, as a measure of how strongly the portfolio value reacts to an increase in informational disorder [61]. It is also treated as an indicator based on the asymmetry of the frequency of positive and negative price changes, used to identify crisis states [62], and as a measure of energy in the active zone of the order book, strongly correlated with liquidity [63].

The Crofton–Steinhaus length and the transactional entropy of the intersection distribution describe two complementary aspects of trajectory complexity, namely the density of decision dilemmas and their disorder. A natural step is therefore to introduce a quantity that combines these two aspects into a single operational scale, a measure of the sensitivity of uncertainty to the intensity of transactional activity. This role is played by the trajectory temperature.

### 11.1. Definition of the Temperature of a Financial Instrument

We draw a line $\left(p^{*},\phi^{*}\right)$ from the invariant measure $\mu_{p}$ (32) normalized on the set of lines I(γ) intersecting γ and define the random variable:

$$N:={\overset{ˇ}{f}}_{\gamma }\left(p^{*},\phi^{*}\right).$$

Assume that the distribution of the number of intersections *N* depends on a parameter θ>0. The mean number of intersections and the entropy are functions of θ:

$$E\left(\theta \right):=\langle N\rangle \left(\theta \right)=\sum_{k}k\cdot P_{\theta }\left(k\right),$$

$$S\left(\theta \right)=-\sum_{k}P_{\theta }\left(k\right)\log P_{\theta }\left(k\right).$$

where $P_{\theta }\left(k\right):=P_{\theta }\left(N=k\right)$. We then define the temperature *T* of the trajectory of a financial instrument as (cf. the classical definition of temperature [64]):

(35) $$\frac{1}{T}:=\frac{dS/d\theta }{dE/d\theta },$$

which we interpret as a measure of the sensitivity of entropy to changes in the mean number of intersections. Here E(θ) denotes an expectation value and serves as a purely formal analogue of energy in our maximum-entropy framework, rather than a physical internal energy.

It is worth emphasizing that, in our framework, temperature does not describe the direction of price movements, but rather properties of the intersection-count distribution through the relationship between the entropy *S* and the mean number of intersections *E*. In particular, temperature reflects the sensitivity of entropy to changes in *E*, i.e., how rapidly the disorder of the intersection distribution increases as the intensity of transactional activity grows.

Consequently, during near-discontinuous crashes, when the dominant direction of motion is unambiguous, the distribution of intersection counts will certainly become narrower, and the entropy, in our geometric setting, will decrease substantially. This may keep the temperature at a similar level or lower it. In contrast, during violent and turbulent market events, when declines are accompanied by numerous rebounds and strong irregularities of the trajectory, the number of intersections will increase significantly, and the entropy will rise. Under such conditions, one may expect a transition into a high-temperature regime, understood as a state of relative saturation with uncertainty, in which further increases of *E* translate only into small changes of *S*. It is also worth noting that in standard approaches to financial risk, risk itself refers directly to forecast uncertainty. In our approach, this aspect of the market is inferred only indirectly, through measurements of entropy and temperature.

### 11.2. Maximum Entropy Distribution and the Low-Temperature Limit

Maximizing

$$S\left(\theta \right)=-\sum_{k}P_{\theta }\left(k\right)\;\log P_{\theta }\left(k\right)$$

under the constraints

$$\sum_{k}P_{\theta }\left(k\right)=1,\;\sum_{k}k\;P_{\theta }\left(k\right)=\bar{E}\;\left(\mathrm{constant}\right)$$

and using the standard method of Lagrange multipliers leads to the family

$$P_{\theta }\left(N=k\right)=\frac{e^{-\theta k}}{Z(\theta )},\;Z\left(\theta \right):=\sum_{j\in K}e^{-\theta j},$$

where $K\subseteq N_{0}=\left\{0,1,2,\ldots \right\}$ is the set of admissible values of *k*.

Since

$$\frac{dZ}{d\theta }=\sum_{k}\left(-k\right)\;e^{-\theta k}=-\sum_{k}k\;e^{-\theta k}=-\;Z\left(\theta \right)\;\sum_{k}k\;\frac{e^{-\theta k}}{Z(\theta )}=-\;Z\left(\theta \right)\;E\left(\theta \right),$$

we have

$$\;E\left(\theta \right)=-\frac{d}{d\theta }\log Z\left(\theta \right).$$

Entropy has the following form

$$\begin{array}{cc} S(\theta )\; & =-\sum_{k}P_{\theta }\left(k\right)\;\log P_{\theta }\left(k\right)=-\sum_{k}P_{\theta }\left(k\right)\;\left(-\theta k-\log Z(\theta )\right)\\ \; & =\theta \sum_{k}kP_{\theta }\left(k\right)+\log Z\left(\theta \right)\sum_{k}P_{\theta }\left(k\right)=\theta \;E\left(\theta \right)+\log Z\left(\theta \right). \end{array}$$

Differentiating the above expression with respect to θ

$$\begin{array}{cc} \frac{dS}{d\theta }\; & =E\left(\theta \right)+\theta \;\frac{dE}{d\theta }+\frac{Z^{\prime }\left(\theta \right)}{Z(\theta )}=E\left(\theta \right)+\theta \;\frac{dE}{d\theta }-E\left(\theta \right)=\theta \;\frac{dE}{d\theta }. \end{array}$$

and using the definition (35), we obtain

$$T=\theta^{-1}\;.$$

We will show that when temperature vanishes, entropy also vanishes.

$$\begin{array}{cc} \frac{dE}{d\theta }\; & =\frac{d}{d\theta }\sum_{k}k\;P_{\theta }\left(k\right)=\sum_{k}k\;\frac{dP_{\theta }\left(k\right)}{d\theta }=\sum_{k}k\;P_{\theta }\left(k\right)\left(E(\theta )-k\right)\\ \; & =E\left(\theta \right)\sum_{k}k\;P_{\theta }\left(k\right)\;-\;\sum_{k}k^{2}P_{\theta }\left(k\right)\\ & =-\;{\mathrm{Var}}_{\theta }\left(N\right)\leq \;0. \end{array}$$

In particular, for θ>0, we have $\frac{dS}{d\theta }\leq 0$, so S(θ) is a nonincreasing function.

Let $k_{\min}:=\min K$ and $\Delta_{k}:=k-k_{\min}\geq 0$. Then

$$Z\left(\theta \right)=e^{-\theta k_{\min}}\left(1+\sum_{\Delta_{k}>0}e^{-\theta \Delta_{k}}\right)=e^{-\theta k_{\min}}\left(1+R(\theta )\right),\;R\left(\theta \right):=\sum_{\Delta_{k}>0}e^{-\theta \Delta_{k}}.$$

Further,

$$\begin{array}{cc} E(\theta )\; & =\frac{\sum_{k}ke^{-\theta k}}{Z(\theta )}=\frac{\sum_{k\in K}\left(k_{\min}+\Delta_{k}\right)\;e^{-\theta (k_{\min}+\Delta_{k})}}{e^{-\theta k_{\min}}\left(1+R(\theta )\right)}\\ \; & =\frac{e^{-\theta k_{\min}}\;\left[k_{\min}\left(1+R(\theta )\right)+\sum_{\Delta_{k}>0}\Delta_{k}e^{-\theta \Delta_{k}}\right]}{e^{-\theta k_{\min}}\left(1+R(\theta )\right)}\\ \; & =k_{\min}+\frac{\sum_{\Delta_{k}>0}\Delta_{k}e^{-\theta \Delta_{k}}}{1+R(\theta )}=:k_{\min}+Q\left(\theta \right). \end{array}$$

where Q(θ)≥0. Since for each $\Delta_{k}>0$ we have $e^{-\theta \Delta_{k}}\to 0$ as θ→∞, it follows that

$$R\left(\theta \right)\underset{\theta \to \infty }{\to }0,\;Q\left(\theta \right)=\frac{\sum_{\Delta_{k}>0}\Delta_{k}\;e^{-\theta \Delta_{k}}}{1+R(\theta )}\underset{\theta \to \infty }{\to }0.$$

Finally,

$$S\left(\theta \right)=\theta \;\left(k_{\min}+Q\left(\theta \right)\right)+\log\;\left(e^{-\theta k_{\min}}\left(1+R\left(\theta \right)\right)\right)=\underset{\to \;0}{\underbrace{\theta Q(\theta )}}+\underset{\to \;0}{\underbrace{\log\left(1+R(\theta )\right)}}\underset{\theta \to \infty }{\to }0\;,$$

$$\lim_{T\to 0^{+}}S\left(\theta \left(T\right)\right)=0.$$

This result can be viewed as an analogue of the third law of thermodynamics. Indeed, in the low-temperature limit $T\to 0^{+}$ (equivalently θ→∞, since $T=\theta^{-1}$), the maximum-entropy distribution $P_{\theta }$ concentrates on the minimal admissible intersection number $k_{\min}$, so the uncertainty of the random variable *N* vanishes and S(θ)→0.

Temperature measures the effect of a change in the mean number of intersections *E* on the uncertainty *S* (entropy). At low *T*, a small increase in the mean number of intersections *E* significantly increases the uncertainty *S*, and the trajectory of a financial instrument is informationally sensitive. At high *T*, even a substantial increase in *E* changes *S* only slightly, and the trajectory of a financial instrument is already saturated with uncertainty.

## 12. Conclusions

The present paper is theoretical in nature and aims to formulate and justify a construction linking risk to the length of a financial instrument’s trajectory in a fixed frame of reference. The proposed approach also provides a basis for introducing new notions, such as the geometric volatility implied by the length of order *m*, relative transactional entropy, and trajectory temperature.

The next step is empirical testing and validation on market data. Already at the implementation stage, however, one must make choices that affect the interpretation of the results. This concerns in particular the selection of the frame of reference, i.e., the choice of money and the benchmark instrument, the construction of financial time, the sampling rules for the data, and the selection of parameters related to the length of order *m*. For this reason, the empirical part requires a separate, dedicated study. These studies may be highly important. In this context, it is worth mentioning that Johann Radon, by generalizing Crofton’s integral geometry, provided a precise description of the transform that underlies tomography, yet satisfactory practical results were achieved only after about 50 years of intensive research. Similarly, empirical investigations of risk understood in this way may yield new kinds of information about the market. However, given the analogy above, this is likely to be a long and demanding research program.

It is important to stress that the probabilistic connections between the model and other variables, like temperature or volatility, are based on assuming particular families of distributions for the number of intersections (for example, Poisson or negative binomial). This means that these connections need not be universal and can be tested against empirical data from specific markets. On the other hand, as the current state of the model is purely theoretical, without implementation and empirical verification, we are not yet in a position to evaluate the robustness of the estimators, their vulnerability to noise, or their predictive power. It is also important to mention that information-theoretic (entropic) approaches are more natural for particular classes of financial instruments and time scales, while applying them to other cases may involve additional considerations.

Dominant risk measures in financial mathematics are built on a random variable, most often representing a loss. They provide a statistical picture of changes. They distinguish the structure of the path followed by the rate of return only to a limited extent. Different trajectories may have similar dispersion or a similar tail profile. At the same time, they may generate a different “density” of decision points in time. There is a large literature on measures of trajectory complexity, such as total variation, *p*-variation, roughness, and Hölder-type measures, the Hurst exponent, and fractal tools. These measures formalize irregularity through increment properties and scaling. In practice, however, they can be sensitive to sampling and to data microstructure. Their values depend on the scale of astronomical time at which the process is observed. Reference time, which is part of our model, moves the analysis to a dimensionless axis determined by the benchmark. As a result, we relate the intensity of the measure to the “internal pace of market” rather than to an arbitrary unit such as minutes or days. This improves the comparability of results across periods and across assets.

In the Crofton risk approach, path complexity is not measured by increments or scaling exponents. It is measured by the averaged number of intersections of the trajectory with the family of simple instruments. This can be viewed as a generalization of level crossing counts known from stochastic process theory and econometrics. We do not choose a single threshold. We average the number of intersections over the whole family of lines. This includes all levels and directions. We use a measure that follows from the geometry of the problem. An intersection can be directly interpreted as a potential decision event with respect to a simple instrument. Risk, therefore, becomes a measure of the intensity of the transactional process generated by the path. The introduction of the length of order *m* plays the role of a regularization known from the discussion of the “coastline paradox”. It limits the contribution of micro-oscillations. It also corresponds to the finite decision and execution resolution of a market observer.

Our formalism depends on the choice of a reference frame, that is, on specifying the pair money and benchmark. In this approach, risk is not an absolute property of an asset. It is a property of the agent who evaluates the asset relative to a chosen unit of account and to a chosen reference asset. Different market participants may therefore rationally adopt different benchmarks, because they have different objectives, horizons, constraints, and reference baskets. Consequently, we allow that two reasonable benchmark choices may yield quantitatively different risk values and potentially different risk rankings. Such variability of rankings does not indicate inconsistency of the measure. Rather, it reveals the viewpoint dependence of risk assessment, in the same way that investment performance and risk are reported relative to an adopted benchmark in practice.

At the same time, the construction preserves clear invariance properties within a fixed reference frame. These properties allow one to compare trajectories in a way that is robust to descriptive conventions, such as the choice of astronomical time units. The question of an “optimal” frame has no universal answer in our interpretation, because it depends on the agent’s objective function, utility, and constraints. For this reason, we do not postulate the existence of a single reference frame that would be best in every application. In comparative studies, it is natural to adopt the standard benchmark for a given asset class. In empirical applications, one may also choose the benchmark so as to improve the properties of estimation and to reduce its uncertainty, which may depend strongly on the quality of the available data.

The proposed view of the risk associated with holding a financial instrument is primarily transactional in nature. In this sense, we identify risk with a measure of the possibility of exchanging a financial instrument for a simple instrument (or conversely), that is, with a measure of the possibility of acquiring or disposing of it. A buy-sell transaction is a single event in which two parties participate. Hence, the measure of events of disposing of an instrument is always equal to the measure of events of acquiring it, and it can be expressed through the length of the trajectory of a given instrument, in accordance with Crofton’s idea, that is, as a measure of the set of lines intersecting the trajectory of a financial instrument. We called the risk understood in this way the Crofton risk.

The transactional interpretation of intersections is a potential component of our approach. An intersection point is not identified with an actual transaction. It represents a temptation to exchange, that is, a moment when the observer faces the dilemma of replacing the risky asset with a simple instrument, or conversely. In real markets, only a fraction of such temptations may translate into action, because transaction costs, delays, liquidity constraints, and execution limits can render many of them unprofitable or infeasible. Therefore, we interpret the proposed risk as a measure of the intensity of decision situations generated by the path, rather than as a count of executed transactions.

Additional justification for length as a risk carrier is provided by a scaling argument. Suppose that we have rescaled the metric. In the geometric sense, this operation corresponds to a homogeneous stretching of space, which means that all distances between points increase by the same factor. In such a rescaled space, the scale of fluctuations of a diffusion process also changes. Measures based on squares of deviations grow proportionally to the square of the scale, and therefore the characteristic linear scale of dispersion, interpreted as the typical magnitude of fluctuations, for example, the standard deviation, grows proportionally to the scaling factor itself. Since rescaling the metric structure leads to a proportional rescaling of the typical dispersion of diffusion, risk constructions based on the metric, distances, trajectory lengths, and related quantities, preserve dimensional and interpretative consistency. Risk increases in the same way distances do. In the context of the transactional interpretation presented above, risk increases with the increase in the number, that is, the frequency, of potential opportunities to enter into a buy-sell transaction.

The concept of simple instruments, which is crucial for our model, can be derived naturally from the APR rate. If we reduce all information about an instrument to a single parameter *r*, its graph on the logarithmic scale becomes a line segment, and the overall trajectory is a curve arising as a sequence of such local approximations as *r* changes over time.

The proposed approach is not limited to a geometric interpretation of risk as the length of a financial instrument’s trajectory; it also provides a basis for introducing additional quantities inspired by thermodynamic formalism, such as entropy or temperature, which may reveal subtle features of market structure and dynamics.

It is worth noting that in finance, projective geometry is a convenient tool for describing the market [65], in which portfolios are treated as equivalence classes. This approach is natural because in valuation and market analysis, proportions between quantities are crucial, not their values. As a consequence, we allow for the possibility that the distance between different representations of an economically equivalent state is zero (a pseudometric), and geometric quantities are constructed so as to respect these identifications and the corresponding invariance properties. Defining a metric structure starting from projective space and based on a measure on the space of lines, which we interpret as simple instruments, appears to be a promising direction for further research into market characteristics.

## Data Availability

Data is contained within the article.
